# Effect of Gene Silencing of Translation Initiation Factors eIF(iso)4G and eIF(iso)4E on Sour Cherry Rootstock Resistance to Sharka Disease

**DOI:** 10.3390/ijms24010360

**Published:** 2022-12-26

**Authors:** Lilia Mourenets, Alexander Pushin, Vadim Timerbaev, Tatyana Khmelnitskaya, Eduard Gribkov, Nikita Andreev, Sergey Dolgov

**Affiliations:** 1 The Branch of the Shemyakin-Ovchinnikov Institute of Bioorganic Chemistry of the Russian Academy of Sciences, 142290 Pushchino, Russia; 2 Nikita Botanical Gardens — National Scientific Centre, Russian Academy of Sciences, 298648 Yalta, Russia; 3 All-Russia Research Institute of Agricultural Biotechnology, Russian Academy of Science, 127550 Moscow, Russia; 4 Biological Institute, The National Research Tomsk State University, 634050 Tomsk, Russia

**Keywords:** *Prunus*, clonal rootstock, sharka, plum pox virus, translation initiation factor, RNA interference, gene silencing

## Abstract

Sharka disease, caused by the *Plum pox virus* (PPV), is one of the most harmful, quarantine viral diseases that affect stone fruit crops. The absence of natural resistance to the virus in stone fruits has become a decisive factor for the use of genetic transformation methods to obtain stable forms. The *eIF(iso)4G* and *eIF(iso)4E* genes encode translation initiation factors used in the PPV life cycle. In the presented study, the effect of silencing these genes using the RNA interference method on the resistance of sour cherry rootstock 146-2 plants (*Prunus pumila* L. x *Prunus tomentosa* Thunb) to the sharka disease was studied. Two vectors have been created for the genetic transformation of plants, with self-complementary sequences of the *eIF(iso)4G* and *eIF(iso)4E* gene fragments. The hairpin expression cassette contains a strong promoter of the peach ribulose-1,5-bisphosphate carboxylase/oxygenase (RuBisCo) gene, as well as an intron and terminator of the same gene. We used the pMF1 vector containing recombinase R and a *codA-nptII* gene which makes it possible to obtain intragenic marker-free plants. A successful genetic transformation was carried out by the AGL0 strain of *A. tumefaciens*. Whole leaves of shoots cultivated in vitro were used as a source of explants. Eight independent transgenic lines of rootstock 146-2 were obtained in experiments (sixlines with a hairpin to the *eIF(iso)4G* gene and two lines with a hairpin to the *eIF(iso)4E* gene). Their status was confirmed by the PCR and Southern blotting. The obtained plants were acclimatized in a greenhouse. The silencing of the *eIF(iso)4G* and *eIF(iso)4E* genes in transgenic plants was confirmed by the quantitative PCR. The presence of specific small interfering (si) RNAs was confirmed by the method of Northern blotting. Plants of all transgenic rootstock lines were infected with PPV by the method of grafting with infected buds. Resistance to the PPV infection of the obtained transgenic plants was carried out by using an enzyme immunoassay. The ELISA results showed that silencing the *eIF(iso)4G* gene did not lead to increased resistance while silencing the *eIF(iso)4E* factor gene led to increased resistance to the PPV, and the one line’s plants showed no signs of infection for two years after infecting. The work demonstrates a (promising) approach in which the creation of stone cultures resistant to the plum pox virus can be achieved by suppressing the genes of translation initiation factors in clonal rootstocks.

## 1. Introduction

Sharka disease (plum pox), caused by *Plum pox virus* (PPV), is the most dangerous viral disease for stone fruit producers. It manifests itself as severe symptoms on leaves, flowers, and, more problematic, on fruits, affecting their organoleptic qualities and leading to malformations, necrosis, and sometimes early abscission. As a consequence, infected fruits are often unsalable. Sharka was first discovered on plum trees in Bulgaria in 1917–1918 and was recognized as a viral disease by Atanassov. Since then, the virus has spread gradually throughout most of Europe, around the Mediterranean Basin, and in the Near and the Middle East. It has also spread to South and North America, Asia, and Africa [1,2].

PPV is a member of the *Potyvirus* genus in the *Potyviridae* family. It has a single-stranded positive-sense RNA genome of approximately 10 kb. The large open reading frame (ORF) is translated into a 355 kDa polyprotein, which is processed by three virally encoded proteases to 10 mature proteins (P1, HcPro, P3, 6K1, CI, 6K2, VPg, NIa, NIb, CP) [3,4]. A short, overlapping ORF called PIPO appears during the transcriptional slip of the viral RNA-dependent RNA polymerase and is expressed as a product of the P3N-PIPO fusion required for virus movement from cell to cell [5]. PPV is transmitted by grafting and other methods of vegetative propagation and by aphids such as *Aphis spiraecola* and *Myzus persicae* in a non-persistent manner [6]. Aphids can acquire the virus from infected leaves, flowers, or fruits in very short periods of time (seconds to minutes) and can transmit it to new plants within minutes [7].

Under natural conditions, the disease affects plants of the genus *Prunus*, which are used as commercial cultivars and rootstocks. These are plants of apricot (*P. armeniaca*), cherry plum (*P. cerasifera*), wild Chinese peach (*P. davidiana*), domestic plum (*P. domestica*), mahaleb cherry (*P. mahaleb*), *P. marianna*, Chinese plum *(P. mume*), peach (*P. persica*), Japanese plum (*P. salicina*), as well as their interspecific hybrids. Sweet cherries (*P. avium*), sour cherries *(P. cerasus*), and almonds (*P. dulcis*) can be infected occasionally or only with specific strains of PPV. In addition, several ornamental and wild plum species are natural hosts of PPV [3].

Sharka is especially dangerous for apricots, European plums, peaches, and Japanese plums because it can reduce seriously reduce fruit yield and quality. In some cases, losses of susceptible varieties can in some cases be as high as 100% [4]. The costs associated with the disease in many countries include not only direct losses associated with crop and quality losses, quarantine, eradication, and compensatory measures, but also indirect costs associated with preventive measures, inspections, diagnostics and their impact on foreign and domestic trade [4]. The cost of containing sharka worldwide has been estimated to have exceeded €10 billion since the 1970s [2].

There is no anti-virus treatment available to control sharka disease in orchards. Effective methods to control the spread of *Plum pox virus* include growing healthy plants for planting according to a certification system, controlling aphid vectors by regular spraying with aphicides, and destroying diseased trees in orchards. Such methods are being used to contain PPV in several countries (for example, France and Italy) [8].

Given the impossibility of rehabilitating infected plants with chemical treatments, the best approach to control sharka disease is to develop PPV-resistant stone fruit cultivars. Although several resistant genotypes are currently known as potential breeding material [9,10], only cultivars showing a PPV-induced hypersensitive response are considered possible sources for partial tolerance breeding in plums [11].

In the early 1990s, the development and improvement of genetic engineering and plant biotechnology methods made it possible to obtain the first PPV-resistant plums [12]. Transgenic plum plants of the C5 line (later called Honey Sweet) contained the PPV coat protein gene sequence and showed resistance to the virus over several growing seasons [13]. More recent studies have shown that PPV resistance was due to post-transcriptional gene silencing (PTGS) [14]. Later RNA technology has been successfully used to obtain transgenic plants with a high level of resistance to sharka disease [15,16,17,18,19]. In addition to the expression of various fragments of the PPV sequence in transgenic *Prunus* plants, a successful result was also shown by the expression of single-chain antibodies specific to the replicase of the NIb virus [20,21].

Another genetic engineering approach is based on the use of the mechanism of silencing the expression of host genes involved in the life cycle of the virus. Eukaryotic translation initiation factors (eIFs) play a leading role in infection with potyviruses [22]. *Potyviruses* require eIFs such as eIF2Bβ, eIF4E, and eIF4G, and their eIF(iso)4E and eIF(iso)4G isoforms to replicate and spread within the cell. Spontaneous or induced mutations in genes encoding translation factors can cause recessive resistance to this potyvirus infection [23]. A 2020 review [22] reported 19 examples of plant resistance to *Potyviruses* (TuMV; PVY; TEV, etc.), due to recessive mutations in the genes of factors eIF4E, eIF(iso)4E, eIF4G, eIF(iso)4G and eIF2Bβ and eIF3b. Obviously, in order to confer resistance to the *Potyvirus* in a plant, plausible approaches are to suppress the expression of the gene for the translation factor that the virus uses or to edit its sequence to eliminate the interaction between the factor and the virus. For example, in the work of Rodriguez-Hernandez et al., it was shown that transgenic melon plants with the *Cm-eIF(iso)4E* gene silencing acquire resistance to 4 viruses at once [24]. Potato plants acquired resistance to PVY after they expressed RNAi specific for *eIF4E1* and *eIF4E2* [25]. In the work of Rupp et al. [26], on the example of wheat, the acquisition of resistance of transgenic lines with gene silencing of the factor TaeIF4G or TaeIF(iso)4E to *Wheat striped mosaic virus* (WSMV) and *Triticum mosaic virus* (TriMV) was shown. Recently, genome editing technology based on CRISPR/Cas9 has become widespread. Thus, modification with loss of function of the eIF4E or eIF(iso)4E factors led to the emergence of resistance to *Potyviruses* in cucumber [27], *Arabidopsis* [28], tomato [29], cassava [30], etc., plants. Le et al., in 2022, demonstrated the acquisition of tobacco resistance to PVY after modification of eIF4E factors [31].

It is important to note that, the complexity of *Prunus* regeneration and transformation processes are still limiting factors for gene transfer technologies that are highly dependent on the genotype [32]. There are only two works known to develop PPV-resistant plum based on gene silencing of translation initiation factors. For example, in 2013, it was shown that several transgenic lines of the common plum, in which the expression of the translation initiation factor *eIF(iso)4E* was suppressed by RNA silencing, showed resistance to PPV infection [33]. In 2019, it was shown that silencing the expression of translation initiation factor *eIF(iso)4G* in Japanese plum *(P. salicina*) reduces the susceptibility of transgenic lines to *Plum pox virus* [34].

Although this approach is promising, the difficulties caused by the particular complexity of the genetic transformation of *Prunus* cause limits to the application of this method. In this regard, obtaining new transformational events associated with the regulation of the expression of eukaryotic translation initiation factors remains an urgent and not trivial task.

The aim of our research was to obtain PPV-resistant transgenic plants of the clonal Russian rootstock 146-2 of stone fruit crops (*Prunus pumila L.* x *Prunus tomentosa* Thunb.) using the technique of silencing the expression of the *eIF(iso)4G* and *eIF(iso)4E* genes by RNA interference.

## 2. Results

### 2.1. Creation of Vectors for Plant Transformation in Order to Silence the Genes of Translation Initiation Factors

**Figure 1:**
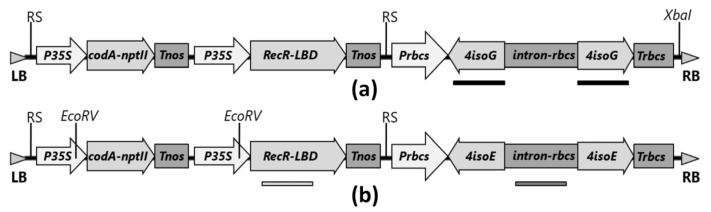
Scheme of the T-DNA region of vectors pMF-R6-iso4G (**a**) and pMF-R6-iso4E (**b**) used for the transformation of rootstock plants. RS, recombination site; RB and LB, right and left border of T-DNA, respectively; P35S, CaMV35S promoter; *codA-nptII*, translational fusion gene for positive (*nptII*) and negative (*codA*) selection; Tnos, nopaline synthase terminator; *RecR-LBD*, translational fusion of Recombinase R-LBD; Prbcs, Trbcs, and intron-rbcs, ribulose-(1.5)-bisphosphate carboxylase/oxygenase small subunit gene promoter, terminator and intron, respectively; *4isoG*, *eIF(iso)4G* eukaryotic initiation translation factor gene (fragment 0.6 kb); *4isoE, eIF(iso)4E* eukaryotic initiation translation factor gene (fragment 0.6 kb). *Xba*I and *Eco*RV, position of the restriction sites for which the DNA was digested for the Southern blot assay. Black, dark gray, and gray stripes indicate the place of hybridization of the probes.

For RNA interference silencing of the translation factor genes *eIF(iso)4G* and *eIF(iso)4E*, we created binary vectors with inverted repeats of target genes. When creating binary vectors, it was taken into account that the expression cassette providing RNA interference should be under the control of a promoter, terminator, and intron of plant origin. These regulatory sequences were cloned from the genome of the peach cultivar Rodzinka. A 578 bp fragments of the *eIF(iso)4G* gene (GenBank no. XM_007204216) conserved among stone fruit species and *eIF(iso)4E* (GenBank no. XM_007227439) gene were amplified from peach cDNA. The assembly of each expression cassette was carried out in an intermediate vector of the pUC18 type. Next, the finished cassette was subcloned into the pMF1 binary vector [35]. The pMF1 vector contains the Recombinase R gene fused to the ligand-binding domain of the glucocorticoid receptor and the bifunctional selective *CodA-nptII* gene encoding cytosine deaminase–neomycin phosphotransferase II. Both expression cassettes are flanked by recombination sites (RS). This binary vector makes it possible to first select for kanamycin, and then to induce excision of the selective marker (*nptII*) and the genes necessary for this process (everything between the RS sites in T-DNA) from the genome of the transgenic plant. The resulting binary vectors were named pMF-R6-iso4G and pMF-R6-iso4E for *eIFiso4G* and *eIFiso4E* genes, respectively (see Figure 1.)

### 2.2. Agrobacterium-Mediated Transformation of Clonal Rootstock and Production of Putative Transgenic Plants

**Figure 2:**
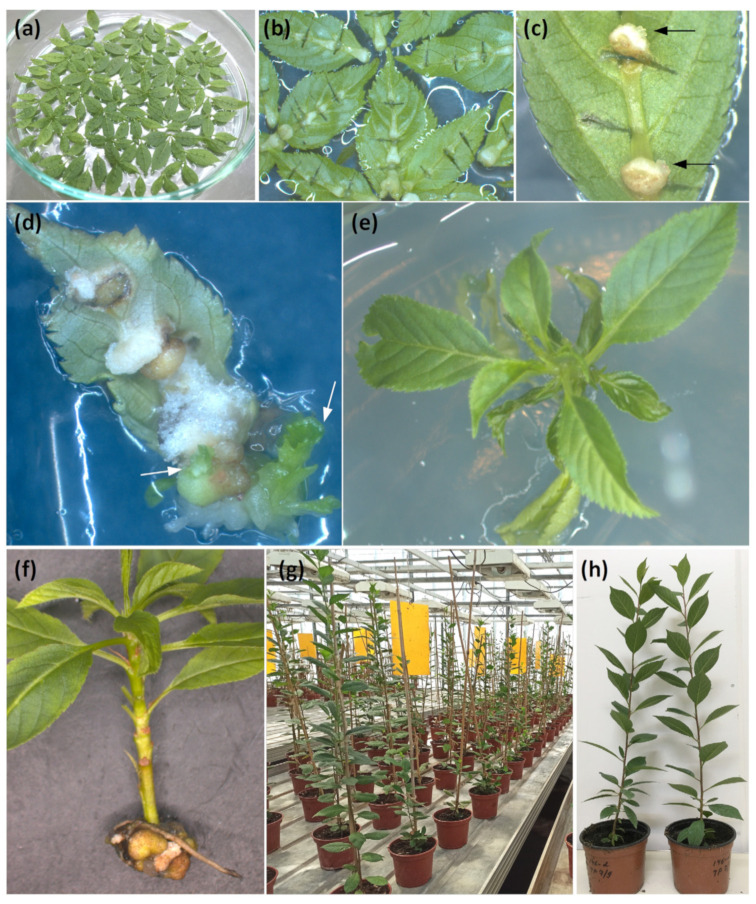
The generation of transgenic 146-2 rootstock plants. (**a**) Rootstock leaf explants in a Petri dish after co-cultivation with *Agrobacterium Tumefaciens;* (**b**) Callus induction on the leaf explants; (**c**) buds formation on the leaf explants of 146-2 rootstock after 7 days in darkness (marked with arrows); (**d**) formation of leaf-like cluster (marked with arrows); (**e**) elongated transgenic shoot; (**f**) rooted rootstock shoot on a medium with kanamycin; (**g**) establishment of transgenic plum rootstock in the greenhouse; (**h**) transgenic plants of rootstock 146-2 before PPV infection experiment.

For each transformation experiment we took 200–500 leaves (explants) and cut them across the midrib several times, as shown in Figure 2a. After 7–10 days, numerous calliappeared on the midrib at the incision sites, as shown in Figure 2c. About half of them died within a month, the rest continued to proliferate, including embryogenic ones, which regenerated shoots as in Figure 2c. Regeneration continued for 2 months from the beginning of transformation. After that period, the calli grew old and did not produce any new regenerants. Shoots proliferating on kanamycin (Figure 2f) were propagated and rooted as in Figure 2f and adapted to greenhouse conditions for growing and research.

Totally, eight agrobacterium-mediated transformations were carried out with the pMF-R6-iso4G vector, as many as with the pMF-R6-iso4E vector. The number of selected kanamycin-resistant clones varied from 0 to 3 in each experiment for the pMF-R6-iso4G. While for the pMF-R6-iso4E vector, eight experiments were managed, and only two of them managed to obtain one plant each. The transformation efficiency (the ratio of the number of resulting kanamycin-resistant clones to the initial number of explants, expressed as a percentage) was from 0 to 0.69%.

### 2.3. Molecular Analysis of Putative Transgenic Rootstock 146-2Plants

**Figure 3:**
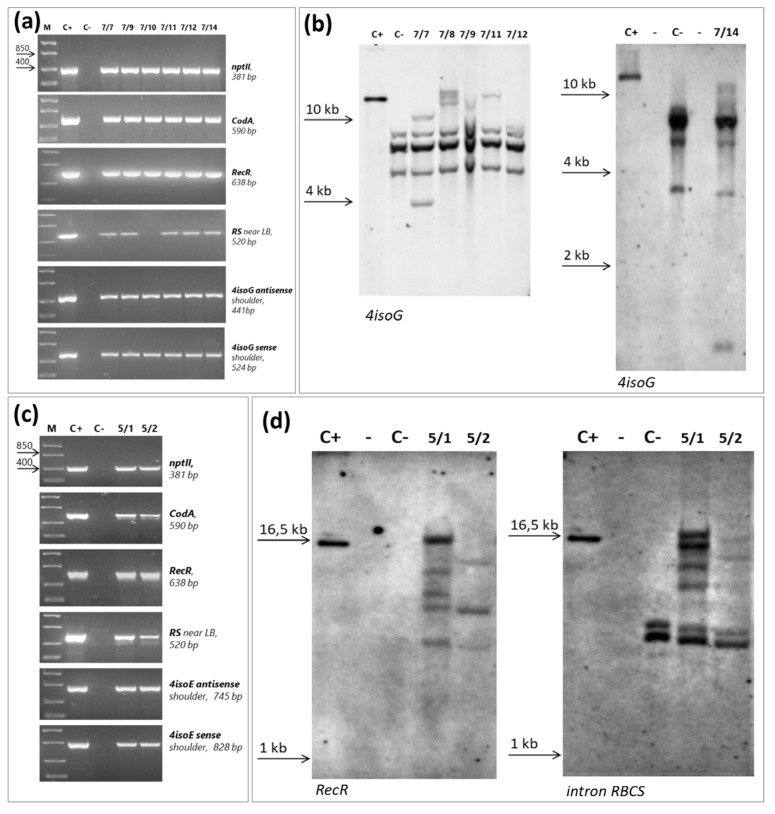
Analysis of rootstock lines 146-2 selected on kanamycin after agrobacterium transformation with vector pMF-R6-iso4G (**a**,**b**) and vector pMF-R6-iso4E (**c**,**d**) by PCR and Southern hybridization. (**a**,**c**) PCR analysis; m- molecular size marker (850, 400 base pairs of DNA); C+—positive control (pDNA); C−—negative control (gDNA of non-transformed plant of 146-2 rootstock); 7/7, 7/9, 7/10, 7/11, 7/12,7/14 and 5/1, 5/2—numbers of analyzed lines; *nptII*, *CodA*, *RecR*, RS, *4isoG*, *4isoE*—the analyzed nucleotide sequences (see Figure 1) and the size of the expected PCR products; (**b**,**d**) Southern blot analysis of transgenic rootstock lines; ***4isoG***The hybridization result of the probe to *eIF(iso)4G* gene followed by digesting the DNA with *Xba*I; ***RecR***the hybridization result of the probe to Recombinase R gene followed by digesting the DNA with *Eco*RV; ***intron RBCS***the hybridization result of the probe to the intron of ribulose-(1.5)-bisphosphate carboxylase/oxygenase small subunit gene followed by digesting the DNA with *Eco*RV.

To confirm the transgenic nature of the transformants, total DNA was isolated from rootstock plants that had taken root and grew well on kanamycin. First, the DNA was checked for the absence of contamination by agrobacteria. For this, primers specific to the region of *virB* virulence located on the Ti plasmid of *Agrobacterium* were used. DNA, in which the region of virulence was not detected, was used to check out the transferred genes using PCR analysis. The result of PCR analysis is shown in Figure 3a,c. Among the rootstock clones selected on kanamycin, which were obtained using the pMF-R6-iso4G vector, clones 7/7, 7/9, 7/11, 7/12, and 7/14 contained the complete T-DNA sequence of this vector (Figure 1a and Figure 3a). Line 7/10 did not contain one RS site for recombinase. This line proved unsuitable for the prospect of inducing the excision of a selectable marker to produce an intragenic plant. Partial integration of T-DNA occurred in the genome of the 7/8 line, resulting in the absence of a hairpin (data not shown). This line was withdrawn from further experiments.

Additionally, the transgenic status of the selected lines was confirmed by Southern blot hybridization (Figure 3b). The probe used for hybridization was specific for the *eIF(iso)4G* sequence, so specific bands are detected in the non-transgenic control too. According to the hybridization profile, all lines were the result of independent transformational events. The number of copies of the transgenic insert varied from one to three (Figure 3b). Thus, as a result of the agrobacterial transformation of rootstock 146-2 with the pMF1-R6-iso4G vector, 6 transgenic lines were obtained.

Rootstock lines 5/1 and 5/2, selected on kanamycin, which were obtained using the pMF1-R6-iso4E vector, contained the complete T-DNA sequence of this vector (Figure 1a and Figure 3c). Southern hybridization confirmed the transgenic status of these lines and the independence of transformation events (Figure 3d). Both clones contained multiple inserts of the transgene. As a result of the agrobacterial transformation of rootstock 146-2 with the pMF1-R6-iso4E vector, 2 transgenic lines were obtained.

### 2.4. Analysis of eIF4isoG and eIF4isoE Genes Expression in Transgenic Clones of Rootstock 146-2

The selected transgenic plants of the rootstock, clones 7/7, 7/9, 7/10, 7/11, 7/12, 7/14, 5/1, and 5/2 as well as the non-transformed control plants were propagated in vitro, adapted in the greenhouse conditions and were cultivated 3 months in the greenhouse. We did not notice any phenotypic differences between control (wild type) plants and the plants of transgenic clones during their growth all this period. Then, total RNA was isolated from the young leaves of the transgenic and control plants of the rootstock, and cDNA was synthesized, which was used for analysis.

**Figure 4:**
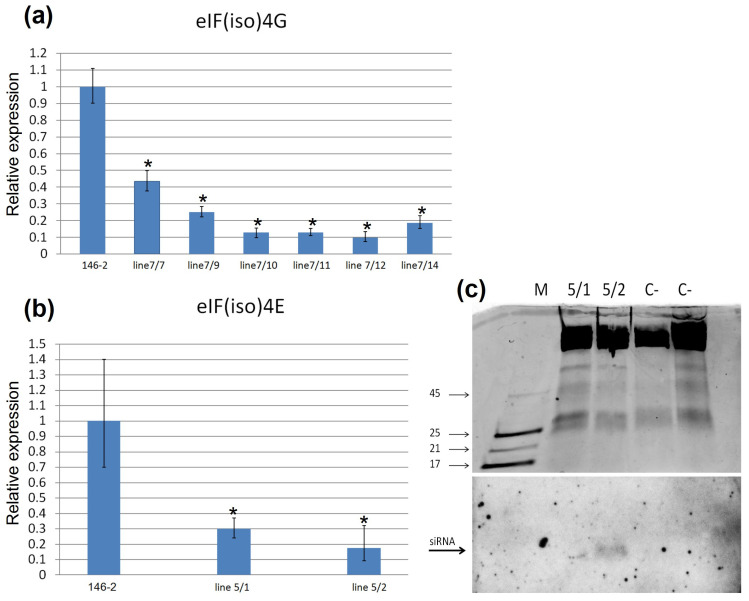
Relative expression of *eIF(iso)4G* and *eIF(iso)4E* genes and siRNAs analysis of rootstock plants carrying the hairpin construct. Reduction in the levels of *eIF(iso)4G*(**a**) and *eIF(iso)4E* (**b**) transcripts quantified by reverse transcription quantitative polymerase chain reaction (RT-qPCR). (**c**) Northern blot of transgene-derived siRNAs in transgenic rootstock lines 5/1 and 5/2. (**top**) The electropherogram of RNA preparations isolated from transgenic lines 5/1 and 5/2 and untransformed control (C−) in 15% PAAG with 7M urea after staining with ethidium bromide; (**bottom**) the hybridization result of the probe to *eIF(iso)4E* gene. M—marker (17, 21, 25, and 45 nucleotides). * *p* ≤ 0.05 relative to control 146-2 in accordance with Kruskal–Wallis test.

The expression levels of the targeted rootstock *eIF(iso)4G* and *eIF(iso)4E* genes in transgenic lines were measured by reverse transcription quantitative polymerase chain reaction (qRT-PCR). The analysis with primers specific for the fragment of rootstock *eIF(iso)4G* gene showed a reduction of the accumulation level of *eIF(iso)4G* mRNAs in all transgenic lines (7/7-7/14) compared with the control rootstock 146-2 plants (Figure 4a). The level of accumulation of mRNA of the *eIF(iso)4G* gene decreased from about 2 to 10 times relative to the control, depending on the transgenic line. The analysis with primers specific for the fragment of rootstock *eIF(iso)4E* gene showed a reduction of the accumulation level of *eIF(iso)4E* mRNAs in 5/1 and 5/2 transgenic lines compared with the control rootstock 146-2 plants (Figure 4b).The level of mRNA accumulation of the *eIF(iso)4E* gene decreased by about three times in the 5/1 line, and in the 5/2 line by almost six times compared to the non-transgenic control.

To assess whether the presence of reduced mRNA levels of the *eIF(iso)4G* and *eIF(iso)4E* genes in transgenic lines are due to the RNA silencing, the accumulation of hairpin-derived small interfering RNAs (siRNAs) was analyzed by Northern blot. Blotting of samples of low molecular weight RNAs extracted from the greenhouse-grown WT, 5/1, and 5/2 plants revealed a siRNA accumulation only in the sample of line 5/2 (Figure 4c). Since no visible bands were found in extracts of WT plants, this observation prompts us to conclude that the presence of small RNAs of 25 nt in the analyzed 5/2 line is the consequence of the degradation of hairpin RNAs due to RNAi silencing. In samples of small RNAs isolated from plants of lines 7/7–7/14, small interfering RNAs specific to the *eIF(iso)4G* gene could not be detected.

### 2.5. Analysis of Plant Resistance of Transgenic Rootstock 146-2 Lines to PPV

**Figure 5:**
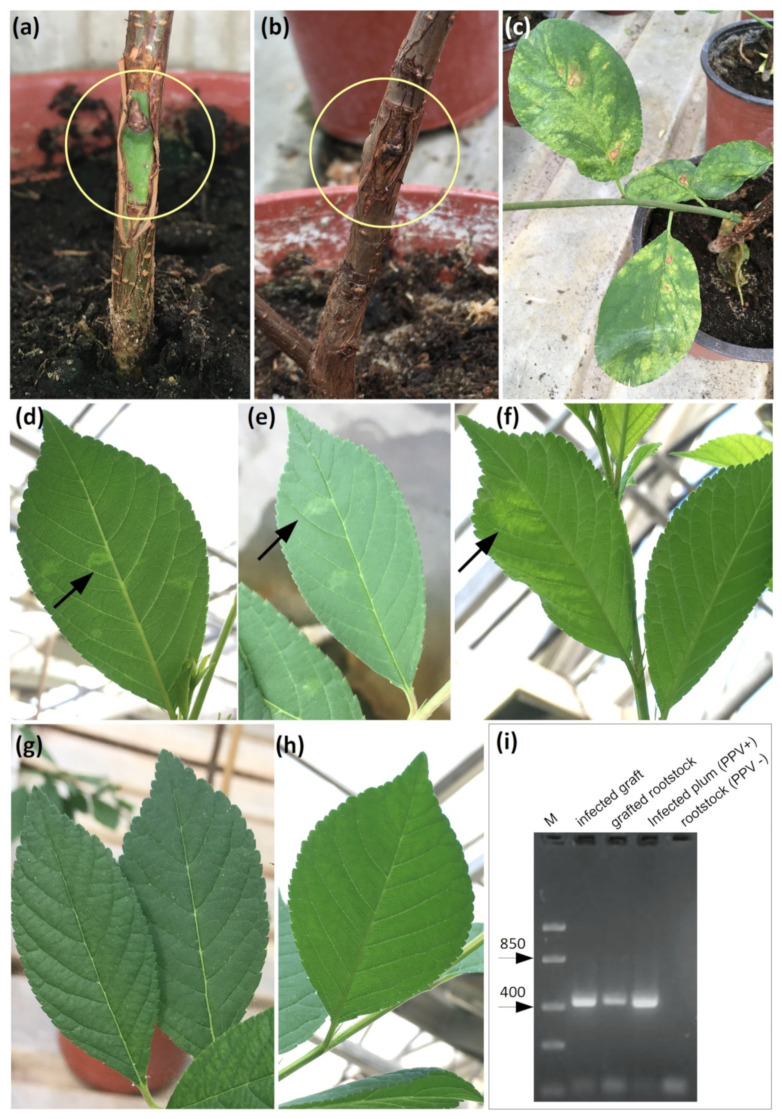
Infection of rootstock 146-2 plants with the *Plum pox virus* in a greenhouse and monitoring the development of the infection. (**a**) T-Budding of a plant of rootstock 146-2 with a plum bud infected with PPV (inside the yellow circle); (**b**) rootstock stem with bud after dormant stage (90 days at 6 degrees) (inside the yellow circle); (**c**) grafting on a rootstock showing signs of infection with a virus (PPV) developed from an infected bud during the first growing season; (**d**,**e**,**f**) signs of PPV infection on rootstock leaves after infection by grafting in the first year of vegetation; (**g**,**h**) leaves of rootstock 146-2, which was not infected with PPV; (**i**) detection PPV by RT-PCR analysis of RNA isolated from infected plum plants—virus donor (PPV+), infected graft, grafted rootstock, non-infected rootstock (PPV−). Amplification of 442 bp fragment of *PPV HC-Pro* gene.

To determine the possible resistance of rootstock transformants, we took 4–5 plants of each clone and grafted them with buds from a diseased plum tree, 1 bud for each plant. Budding was carried out in August 2020 and 2021. The stem section with the grafted bud was wrapped with polyethylene tape for 3 weeks. Next, the tape was removed, and the condition of the grafts was assessed. All grafted kidneys looked viable along with the transferred cortex as shown in Figure 5a. A total of 54 rootstock plants were grafted. After leaf fall, the grafted trees were kept at rest in a greenhouse at 6 °C for 3 months. After that, at the beginning of the period of sap flow, the condition of the vaccinations was again examined. All vaccinations looked viable, as in Figure 5b.

At the beginning of the growing season, after flowering and the appearance of leaves, the manifestation of symptoms was observed on the leaves developed on the shoots from grafted buds (Figure 5c) and on the leaves of the rootstock plants (Figure 5d, e, f) compared with the leaves of uninfected plants (Figure 5g,h). Infection was also confirmed by RT-PCR analysis (Figure 5i). In March, a DAS-ELISA test was carried out to determine the infestation of plants for each clone and for control (wild-type) plants.

**Figure 6:**
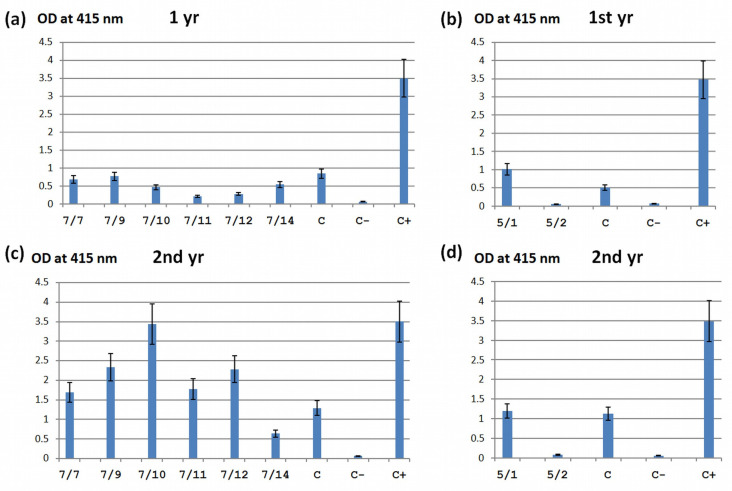
DAS-ELISA analysis of infected transgenic rootstock 146-2 plants after 1 (**a**,**b**) and 2 (**c**,**d**) years from inoculation of PPV. (**a**,**c**) Detection of the virus in transgenic lines (7/7–7/14) containing the *eIFiso4G* gene silencing construct. (**b**,**d**) Detection of the virus in transgenic lines (5/1 and 5/2) containing the *eIFiso4E* gene silencing construct. The rootstock plants 146-2 were not infected with PPV (C−). The rootstock plants 146-2 (wild type) infected with PPV (C). Positive control on PPV from the BIOREBA kit (C+).

All transgenic clones were analyzed using the BIOREBA kit to determine their degree of sensitivity or resistance to the virus. Clones with silenced gene *eIF(iso)4G* showed sensitivity to PPV as early as the first year after infection. According to the test, the clones 7/10, 7/11, and 7/12 gave a signal slightly lower than the non-transgenic control, the clones 7/7, 7/9, and 7/14 showed a signal approximately at the control level (Figure 6a).

When analyzing transgenic plants with *eIF(iso)4E* gene silencing, the 5/1 clone was susceptible, and the 5/2 clone showed a signal at the level of uninfected control plants, as shown in Figure 6b.

In the second year after infecting plants with *eIF(iso)4G* silencing, the signal level for the clone 7/14 remained approximately the same, while for the rest it significantly increased and even exceeded the values of the non-transgenic control (Figure 6c). The remaining clones of this vector showed an increased signal, even higher than that of the non-transgenic control.

The plants transformed with the *eIF(iso)4E* vector and showing sensitivity to the virus (the 5/1clone) gave a signal approximately at the level of wild-type plants, as can be seen in Figure 6d. The signal of the plants of the 5/2 clone remained almost at the level of the uninfected control and corresponds to healthy plants according to this test (Figure 6d). Thus, according to the tests performed, of all the clones for both vectors, only 5/2 turned out to be tolerant to PPV for a period of 2 years after infection. We considered this clone to be promising for the excision of selectable marker sequences and the sites necessary for this process to obtain intragenic plants.

## 3. Discussion

In this work, we studied the effect of silencing the expression of genes encoding translation initiation factors eIF(iso)4E and eIF(iso)4G in clonal rootstock 146-2 on resistance to plum pox virus in a representative of the genus *Prunus*. Initially, it was shown in *Arabidopsis* that PPV infection of plants of this species is associated with the *eIF(iso)4E* gene [36]. Additionally, Nicaise et al. [37] found that the translationally nonfunctional *Arabidopsis eIF(iso)4G1* mutant is resistant to PPV, but not to its knockout counterparts *eIF(iso)4G2* and *eIF4G*. Later work was presented [33] showing that eIF(iso)4E (unlike eIF4E) is involved in PPV resistance in plums (*Prunus domestica*). Transgenic plum lines with RNAi-silencing of the eIF(iso)4E factor gene were characterized by increased resistance to PPV compared to lines with RNAi-silencing of the eIFiso4E factor and non-transgenic control. In this study, the decrease in the expression level of the *eIF(iso)4E* gene when using virus 35S promoter was from 4 to 8 times, which is comparable to our result when using plant promoter from Rubisco gene (3–6 times). This was enough for the emergence of resistance to the PPV.

Using another representative of the genus *Prunus*, the Japanese plum, the authors showed in 2019 that suppression of the *eIF(iso)G11* gene by RNA interference induces resistance to the PPV [34]. Only one transgenic line displays a reduction in *eIF(iso)4G11* transcript (six times relative control) whereas all the other transgenic were chimeras. In lines obtained by us, a decrease in the expression level of the target gene was found after several cycles of vegetative propagation. For the *eIF(iso)4G* transcripts the expression level was 2–10 times lower relative to control plants. Since none of the six transgenic lines showed resistance, the question of the participation of the *eIF(iso)4G* gene in the spread of PPV infection remains open. The authors also suggest that down-regulation of the eIFiso4E factor is fatal to plum trees because they failed to regenerate transgenic plants with the eIF(iso)4E-specific hairpin. This assumption does not agree with the data obtained in previous studies. The papers [38,39,40,41] show that mutant plants lacking either eIF4E or eIF(iso)4E do not show phenotype changes compared to wild-type plants. This suggests some degree of functional redundancy between the two isoforms of factors.

In the present work, we have created two genetic constructs for silencing the genes of the translation initiation factors eIF(iso)4E and eIF(iso)4G using the RNA interference mechanism. Nucleotide sequences of peach (*Prunus persica*) factors available in databases were used to produce the hairpin construct. The promoter, terminator, and hairpin intron were also cloned from this species. In this way, expression cassettes were constructed to generate RNA interference, allowing to obtain intragenic plants as a result. To transfer the expression cassette into the plant genome, we used the pMF1 binary vector [35], which contains T-DNA recombinase R under the control of an inducible promoter, which, after selection of transgenic plants on kanamycin (in our case), induces the removal of the selective marker in transgenic lines. This system was successfully applied in cultures such as strawberries, apples, and pears [35,42,43,44]. We have previously successfully used this design to obtain a marker-free apple tree and tomato plants [45,46].

The constructed pMF-R6-iso4E and pMF-R6-iso4G vectors can be used to transform different representatives of *Prunus*. Dwarf and cold-resistant clonal rootstock 146-2 for plum, apricot, and cherry was used in the work. The rootstock was obtained at Siberian Horticultural Research Institute by the hybridization of sand cherry (*Prunus pumila* L.) and downy cherry (*Prunus tomentosa* Thunb.). Previously, we developed protocols for regeneration and agrobacterial transformation for this rootstock [47,48]. Anyway, the process of obtaining transgenic lines of this rootstock turned out to be a complex and lengthy process, as is often the case with representatives of the genus *Prunus* [49]. In contrast to the work [34], we managed to obtain transgenic lines with both the pMF-R6-iso4G and pMF-R6-iso4E constructs. We confirmed the transgenic nature of the resulting rootstock lines and the independence of transformation events by Southern hybridization. All lines (six based on the pMF-R6-iso4G vector and two based on pMF-R6-iso4E) were characterized by a decrease in the level of mRNA accumulation of the corresponding translation initiation factor. However, we were able to detect specific siRNAs only in plants of the 5/2 transgenic line obtained using the pMF-R6-iso4E vector. Probably, the largest siRNA pool was in the 5/2 lineage and was sufficient for the sensitivity of the small RNA detection method we used. All obtained lines were infected under protected soil conditions with the PPV-M virus. During two growing seasons, the development of the infection was observed visually, and the presence of the virus was also determined by DAS-ELISA. As a result of the experiments, all lines with the silence of the *eIF(iso)4G* gene were infected with PPV in the second year. At the same time, plants of one of the two lines with eIF(iso)4E factor silencing were resistant to PPV in the second year after infection.

In the future, we plan to use an approach that makes it possible to create (develop) stone fruit cultures resistant to the plum pox virus by silencing the genes of translation initiation factors in clonal rootstocks. An additional feature of our study is the possibility of obtaining an intragenic line of clonal rootstock resistant to PPV by recombinase-induced removal of selective and marker genes.

## 4. Materials and Methods

### 4.1. Plant Material

Dwarf and cold-resistant clonal rootstock 146-2 for plum, apricot, and cherry was used in the work. The rootstock was obtained at Siberian Horticultural Research Institute by hybridization of sand cherry (*Prunus pumila* L.) and downy cherry (*Prunus tomentosa* Thunb). Aseptic culture of 146-2 was recovered from the young shoots of adult trees growing in the greenhouse as described earlier [47]. Plant shoots were propagated onmedia with MS macrosalts [50] with DKW micronutrients [51] and FeNaEDTA and QL vitamins [52], supplemented by 0.2 mg/L benzylamino-purine (BA), 0.75 mg/L zeatin, 0.15 mg/L gibberellin, 0.04 mg/L indole-3-butyric acid. The medium contained 30 g/L sucrose and 7 g/L agar; pH was 5.8. Shoots were passed onto fresh medium every 3-4 weeks.

### 4.2. Plasmid Construction

For RNA interference silencing of the translation factor genes *eIF(iso)4G* and *eIF(iso)4E*, we created binary vectors with inverted repeats of target genes. The second intron of the Rubisco gene (ribulose bisphosphate carboxylase small chain) of peach (GenBank no. CM007653) with a length of 368 bp was used as a separating spacer. It was amplified from the genomic DNA of peach cultivar Rodzinka using primers 5′-actgaccggtaggtaagactacttatcaacat-3′ and 5′-actgctcgagcctgtatcaatcaaaatattcg-3′. The gene fragment was cloned in the modified vector pUC18 [53] (restriction sites *Bsh*TI and *Xho*I between the *Xba*I and *Sal*I sites were added to the polylinker) at the *Bsh*TI and *Xho*I sites. In the next step, a 293 bp Rubisco terminator was amplified using primers 5′-actgtcgacatctctcatctctactaagac-3′ and 5′-actgcctgcaggtctggctatcaaacttgaattg-3′. It was cloned in a vector with an intron at the *Sal*I and *Sda*I sites. Then, a 578 bp fragment of the *eIFiso4G* gene (GenBankno.XM_007204216) conserved among stone fruit species was amplified from peach cDNA using primers 5′-actgaccggtaggaccgtgtgttgaagactg-3′ and 5′-actgcccgggctttaaggcgtgattttgggctctcgtc-3′ for anti-sense orientation and 5′-actgctcgagaggaccgtgtgttgaagactg-3′ and 5′-actgtcgacgcgtgattttgg’gctc-3′ for sense orientation. The gene fragments were cloned sequentially, first the left arm (shoulder) at the *Sma*I and *Bsh*TI sites, and then the right arm (shoulder) at the *Xho*I and *Sal*I sites in the vector containing the intron and the terminator. The *eIF(iso)4E* gene (GenBank no. XM_007227439) fragments were cloned according to the same scheme. For their amplification, primers 5′-actgaccggtgacgctgaggaaaatagtggg-3′ and 5′-actgcccgggcttaaggctgatctttcccttttagaatcg-3′ were taken for the left shoulder and 5′-actgctcgaggacgctgaggaaaatagtggg-3′ and 5′-actgtcgacgctgaatctttcccttttagaatcg-3′ for the right shoulder. The last step in the creation of expression cassettes was the cloning of the peach Rubisco gene promoter. A 618 bp gene fragment was amplified using primers 5′-actgagctcggcgcgcccctaccaagtgaagaaacattctatc-3′ and 5′-actacttaagtgctctgtgctctctttagcc-3′ and cloned into intermediate vectors at the *Sac*I and *Bsp*TI sites. The correctness of each cloning step was confirmed by restriction analysis and sequencing. All enzymes used in the work were manufactured by Thermo Fisher Scientific (Waltham, MA, USA). The constructed cassettes were transferred to pMF1 vector (Wageningen Plant Research, Wageningen, Netherlands; [35]) at the *Asc*I and *Sda*I sites. The pMF1 vector contains the recombinase R gene fused to the ligand-binding domain of the glucocorticoid receptor and the bifunctional selective *CodA-nptII* gene encoding cytosine deaminase-neomycin phosphotransferase II. Both expression cassettes are flanked by recombination sites (RS).The resulting binary vectors were named pMF-R6-iso4G and pMF-R6-iso4E for *eIF(iso)4G* and *eIF(iso)4E*genes, respectively.

### 4.3. Agrobacterium Strain for Plant Transformation

We used the supervirulent *Agrobacterium* strain AGL0 to transform the rootstock 146-2 plants [54].

### 4.4. Agrobacterium-Mediated Transformation and Plant Regeneration

Cultivation in vitro and regeneration of shoots from leaf explants were described previously [47].

In transformation experiments, young fully expanded leaves (3rd–6th apical ones) were cut from the shoots and several incisions were made on them across the midrib without cutting through the leaf edges. The explants were placed onto surface of liquid medium in Petri dishes (8 mL/Petri dish) without phytohormones. The medium was supplied with 100 µM of acetosyringone (AS). Every dish contained 100–120 leaves depending on its size. For inoculation, 1.5 mL of night culture of agrobacteria was added to every Petridish with explants after removing of LB medium with antibiotics (50 mg/L rifampicin and 100 mg/L kanamycin) by centrifugation for 5 min at 4500 rpm and resuspending in co-cultivation medium from the dish. The explants were floating abaxial side up on medium surface without immersion into the medium to avoid explants’ death. After 1–1.5 h of co-cultivation, the media were removed by pipetting and replaced with fresh liquid medium completed with 2 mg/L thidiazuron (TDZ), 0.5 mg/L naphthyl-acetic acid, 5 mg/L Ca-pantothenat, and 100 µM AS, for 6–7 days in dark at 24 °C without rotation. To avoid agrobacteria overgrowth the medium was substituted for the fresh one on the 4th day of co-cultivation. After period of cultivation, the explants were dried on sterile filter paper and were placed abaxial side up onto the same medium with agar (7 g/L), cefotaxime (500 mg/L), and kanamycin (10 mg/L) for 10 days in the dark. Then, kanamycin concentration was increased to 20 mg/L and the explants were subcultured every 2 weeks. An amount of 10 mg/L kanamycin in the medium completely disabled shoot regeneration for control explants (data not shown). The number of leaf explants was about 200–500 per transformation.

### 4.5. PCR Analysis and Southern Hybridization

For PCR and Southern blot analysis, the genomic DNA of rootstock 146-2 was isolated from kanamycin-resistant and non-transformed control plants grown in vitro using the method of Dellaporta et al. [55]. The plants were pre-tested for the absence of agrobacterial contamination using primers virBF and virBR that amplify the *virB* gene of *A. tumefaciens* (Table S1). The primer sequences used for the detection of genes from the T-DNA of vectors pMF-R6-iso4G and pMF-R6-iso4E in transgenic plants are presented in Table S1.

Rootstock 146-2 genomic DNA (30 µg) was digested overnight at 37 C with 100 U *Xba*I (for rootstock lines obtained using the vector pMF-R6-iso4G) or *Eco*RV (for rootstock lines obtained using the vector pMF-R6-iso4E) (Figure 1). The DNA of non-transformed rootstock plants digested with *Xba*I or *Eco*RV was used as a negative control. After agarose gel (0.8%) electrophoresis, the digestion products were transferred and immobilized onto Hybond N+ membrane (GE Healthcare, Amersham Bioscience, Amersham, UK) following the manufacturer’s instructions. The DNA probe was constructed by PCR using plasmids pMF-R6-iso4G or pMF-R6-iso4E as the template and primer pairs isoG-F/isoG-R, Trf-up/Trf-low and intRB-up/intRB-low to amplify the sequences of *4isoG*, *RecR* and intron *RB* genes (Table S1). DNA probes (0.6kbp of *4isoG*, 0.6kbp of *RecR* and 0.39kbp of *RB* intron) were labeled with alkaline phosphatase using the AlkPhos Direct Labeling Kit (GE Healthcare, Amersham Bioscience, Amersham, UK). Prehybridization, hybridization (incubated at 60 °C overnight) with alkaline phosphatase labeled probe, and subsequently washings of the membrane were carried out according to the AlkPhos Direct Labeling Kit protocol. Detection was performed using CDP-Star detection reagent following the manufacturer’s directions (GE Healthcare, Amersham Bioscience, Amersham, UK).

### 4.6. RNA Extraction and qPCR Assay

Total RNA was isolated from fresh or frozen leaves of greenhouse plants of clonal rootstock 146-2 using the method described in [56]. To remove DNA residues, the RNA solution was treated with DNase I (Thermo Fisher Scientific, Waltham, MA, USA) in accordance with the manufacturer’s instructions. The middle part of 5th leaf was used for analysis. The cDNA was reverse transcribed from 2 μg of total RNA using MMLV Reverse Transcriptase (Evrogen JSC, Moscow, Russia). RT–qPCR was performed using 5X qPCRmix-HS SYBR+LowROX (Evrogen JSC, Moscow, Russia) with a QuantStudio™ 5 Real-Time PCR Cycler (Thermo Fisher Scientific, Waltham, MA, USA) following the manufacturer’s instructions. The mitochondrial nad5 gene was selected as the internal control [57]. The qPCR analyses were performed with three technical replicates and analyzed using QuantStudio TM Design and Analysis (Applied Biosystems, Waltham, MA, USA; Thermo Fisher Scientific, Waltham, MA, USA). Primers used for qRT–PCR are listed in Supplementary Table S1.

### 4.7. Northern Blot Hybridization

Total siRNA was extracted from young leaves (1 g) using an acid guanidinium thiocyanate–phenol–chloroform extraction method as described in [58,59]. An extracted rootstock 146-2 total RNA (20 μg) was loaded onto 15% polyacrylamide gel containing 7 M urea, and then (after electrophoretic separation in PAAG) electrotransferred onto a membrane (Hybond-N+, GE Healthcare, Amersham Bioscience, Amersham, UK). siRNA bands were probed within the *eIF(iso)4G* or *eIF(iso)4E* 0.6kbp PCR fragments that were labeled with alkaline phosphatase using the Amersham Gene Image AlkPhos Direct Labeling and Detection System (GE Healthcare, Amersham Bioscience, Amersham, UK). Prehybridization, hybridization (incubated at 42 °C overnight) with alkaline phosphatase labeled probe, and subsequently washings of the membrane were carried out according to the AlkPhos Direct Labeling Kit protocol. Detection was performed using CDP-Star detection reagent following the manufacturer’s directions (Amersham CDP-Star Detection reagent, GE Healthcare, Amersham, UK).

### 4.8. Grafting for Virus Inoculation

One-year-old plants of six independent transgenic events, containing eIF4isoG-hpRNA construct, and two independent transgenic events, containing eIF4isoE-hpRNA construct, and non-transgenic rootstock 146-2 plants were inoculated by budding with Plum pox virus, Marcus strain (PPV-M), isolate PS (AJ243957). The infected 1-year-old branches with buds were obtained from Federal State Budget Scientific Institution «North Caucasian Federal Scientific Center of Horticulture, Viticulture, Wine-making» (Krasnodar, Russia). In 2009, the plum of the Startovaya cultivar was infected with budding using this material. In 2020, buds from this infected plum were used to infect rootstock 146-2. Virus inoculation was conducted through the grafting of an individual virus-containing bud onto the transgenic plant. For each independent transgenic line 4 to 5 plants were inoculated. Following grafting, the plants were maintained in the greenhouse at 22–24°C/16–18°C (day/night) for 3 months at natural light, and then the plants went through a cold treatment (about 6°C) for 4 months. The grafting occurred in August 2020 and was repeated in 2022. In the spring of 2021, plants with survived infected buds converted into branches were visually monitored for PPV symptoms after cold treatment.

### 4.9. Analysis of Infected Plants

The grafted young trees were grown in a greenhouse and evaluated every vegetative season for visual symptoms of virus infection. To verify the presence of PPV in the leaves of transgenic and WT plants, molecular analysis was performed in 2021 and 2022.

#### 4.9.1. DAS-ELISA

For every grafted plant, three leaves from different twigs (from their midsections) were collected and used for preparing 100mg tissue samples, then they were homogenized with liquid nitrogen and analyzed.PPV infection was evaluated using a double antibody sandwich enzyme-linked immune-sorbent assay (DAS-ELISA) by the PPV Complete kit 480 of BIOREBA AG (Reinach, Switzerland). DAS-ELISA analysis was performed according to the manufacturer’s instructions according to the following scheme:antiPPV-IgG/antigen (plant extract)/antiPPV-IgG conjugated with alkaline phosphates. Absorbance was measured with an iMark Microplate reader (Bio-Rad, *Hercules*, CA, USA) at 415 nm.

#### 4.9.2. RT-PCR Detection

To examine the virus, total RNAs were isolated from young leaves excised from greenhouse shoots according to [56]. cDNAs were generated from 2μg of the total RNAs using MMLV Reverse Transcriptase (Evrogen JSC, *Moscow*, Russia) and were subjected to PCR. The primers used for the amplification of a 442-bp fragment of HC-Pro gene were PPV1000-s and PPV1300-a (Table S1). PCR products were separated by electrophoresis on 1.2% (*w*/*v*) agarose ethidium bromide gels.

## 5. Conclusions

In this study, we have shown, in the case of the transgenic clone 5/2 of the 146-2 clonal rootstock, that resistance to PPV can be effectively achieved by suppressing the expression of the *eIF(iso)4E* gene using an RNA silencing strategy. The resistance was stable during two cycles of growth and dormancy. The suppression of mRNA synthesis in this line is due to the accumulation of a pool of siRNAs specific to the *eIF(iso)4E* gene sequence. The rootstock line 5/2, which we have obtained, showed resistance to the Marcus strain PPV (isolate PS (AJ243957), as the most dangerous for plum, apricot, and peach.

Taking into account that the inoculating of a plant by budding with an infected graft is the most detrimental method of infection for the recipient plant (the grafting is a constant source of the virus). We assume that in the field, with aphid infection and a natural viral background, plants of the transgenic line 5/2 of the clone rootstock 146-2, can show long-term and stable resistance to the Plum pox virus.

An important distinguishing feature of the obtained line 5/2 is the ability to “transfer” it from transgenic to intragenic status by inducing the removal of a selectable marker, as well as the native origin of the transferred genes.

## Data Availability

Not applicable.

## Supplementary Materials

The following supporting information can be downloaded at: https://www.mdpi.com/article/10.3390/ijms24010360/s1, Table S1: Nucleotide sequences of used primers.
