# Supplementary material for: Effect of Gene Silencing of Translation Initiation Factors eIF(iso)4G and eIF(iso)4E on Sour Cherry Rootstock Resistance to Sharka Disease
Source: Int J Mol Sci. 2022 Dec 26;24(1):360. doi: 10.3390/ijms24010360 (PMC9820581; doi:10.3390/ijms24010360)
Supplement: Supplementary file 1 [file ijms-24-00360-s001.zip › ijms-2095113-supplementary.pdf]

Supplementary Materials

Table S1. Nucleotide sequences of used primers.

**Primers**	**Sequence, 5' → 3'**	**Amplified Fragment, bp**	**Sequence Annealing**	**Were used for the method:**
intRB-up<br>intRB-low	ACTGACCGGTAGGTAAGACTACTTATCAACAT<br>ACTGCTCGAGCCTGTATCAATCAAAATATTCG	389	Intron of Rubisco	Southern Blot
isoG-F<br>isoG-R	ACTGCTCGAGAGGACCGTGTGTTGAAGACTG<br>ACTGTCGACGCGTGATTTTGGGCTC	578	*eIF(iso)4G*	Southern / Northern Blot
isoE-F<br>isoE-R	ACTGCTCGAGGACGCTGAGGAAAATAGTGGG<br>ACTGTCGACGCTGAATCTTTCCCTTTTAGAATCG	578	*eIF(iso)4E*	Northern Blot
virBF<br>virBR	GGCTACATCGAAGATCGTATGAATG<br>GACTATAGCGATGGTTACGATGTTGAC	671	*VirB1*	PCR
nptII-F<br>nptII-R	GCTATGACTGGGCACAACAGACAATC<br>TCCGAGTACGTGCTCGCTCGA	381	*nptII*	PCR
35S-140up<br>CodA-low	CCAACCACGTCTTCAAAGCA<br>AATGCCTTCAAACAGCGTGC	590	*CodA*	PCR
Trf-up<br>Trf-low	ATGCGCAAGGAGGCAGGTCG<br>GCCACACGGGAGACGCCTTC	638	*RecR*	PCR / Southern Blot
RS up<br>35S low	5′-CGATTTGATGAAAGAATGAATTAATG-3′<br>5′-GTGTGT CGTGCTCCACCATG-3′	520	RS site and fragment of CaMV 35S promoter near LB	PCR
4isoG-R<br>intRB-R	TTATTCAAGAGGACACGCTTGAAAGTG<br>ACGACATGTTAAGTTATCAGACTGTAATTCAGAC	441	Fragment of eIF(iso)G antisense	PCR
intRB-F<br>4isoG-R	GTCTGAATTACAGTCTGATAACTTAACATGTCGT<br>TTATTCAAGAGGACACGCTTGAAAGTG	524	Fragment of eIF(iso)G antisense	PCR
intRB-R<br>4isoE2-R	ACGACATGTTAAGTTATCAGACTGTAATTCAGAC<br>GCTGATCTTTCCCTTTTAGAATCGTCAT	745	Fragment of eIF(iso)E sense	PCR
intRB-F<br>4isoE2-R	GTCTGAATTACAGTCTGATAACTTAACATGTCGT<br>GCTGATCTTTCCCTTTTAGAATCGTCAT	829	Fragment of eIF(iso)E sense	PCR
Nad-5-F<br>Nad-5-R	GATGCTTCTTGGGGCTTCTTGTT<br>CTCCAGTCACCAACATTGGCATAA	181	*Nad-5*<br>For qRT–PCR	qPCR
PPV1000-s<br>PPV1300-a	CCAGGAATGAGCGGATTTGTGGT<br>CATGTGAAAATTGTGGATAGTTATCCATCAC	442	mRNA of PPV-M (HC-pro)	RT-PCR
isoG-314-up<br>isoG-full-low	TTGAGGGAAGAAGTGAGGCAG<br>GTCAGTTCCTTCAATCGGCTG	314	eIF(iso)4G	qPCR
isoE-full-up<br>isoE-339-low	ATGGCGACAGAGGTAGCAG<br>AGCACACTCAGGATCCTCC	339	eIF(iso)4E	qPCR
